# Mr. Howship, on Lock Jaw with Tetanus

**Published:** 1809-03

**Authors:** John Howship

**Affiliations:** Member of the Royal College of Surgeons, London, and Assistant Surgeon of the 52d Regiment of Foot. Scarborough


### *To the Editors of the Medical and Physical Journal.*

GENTLEMEN,

SHOULD the following Cases be considered to possess sufficient interest to entitle them to a place in the Medical and Physical Journal, I shall be happy to furnish several others, in which, the effects of amputation and of compression were made the subject of experiment.

---

THE very limited knowledge we at present possess in the successful treatment of Lock Jaw and Tetanus, must impress the mind of every anxious student in pathology, with the conviction that very much remains to be acquired in this department of medical information.

To those who have read of, but have never seen these complaints, the histories already written, and the plans of treatment proposed and described by writers of intelligence, may be sastisfactory; but to such as have been in the habit of watching the various changes in this disease at the bedside of the unfortunate sufferer, it does, and must appear, that we are not yet acquainted with any one medicine or application, upon which, from uniform experience we can rely; nor are we possessed of any remedy that can be exhibited in any confidence of its power to afford relief.

A correct observance of Nature points out that in this, as well as perhaps every other malady to which the human frame is liable, no two individuals are ever affected precisely in in the same manner; although in every case the disease still retains its peculiar and essential characters.

The following cases, as well as many others of the same cast, fell under my own observation, and seem to me worthy of attention.

### CASE I.

*Of Lock Jaw with Tetanus, which terminated fatally.*

On the ever memorable 21st of October, 1805, a lad, 16 years of age, was struck on the hip by a splinter, while engaged on board H. M. S. Colossus. The wound was just opposite to the spine of the right os illium; it was not large, but it was deep, and like the general run of wounds from gunshot, very foul and slow of digestion. Very little pain was felt either in the wound or its neighbourhood.

On the 5th day after, he complained of difficulty in swallowing; this, however, was but the prelude to sufferings infinitely more severe. On the 8th day, when I visited that part of the hospital, he was in dreadful agonies, full of distress and spasm. His jaws by this time were firmly closed. In order to ascertain whether any visible action could be observed in those situations where the pains were described to be most urgent, the clothes were thrown aside, when the whole of the muscles of the abdomen and limbs were found in a state of alternate vibration, falling in succession under the influence of the pain and irritation.

The limb below the wounded part he complained much of, as also of the whole of his breast. A warm bath was proposed and got ready; but it was not without great difficulty and infinite distress, that the patient could be raised from his bed and placed in the bath. He found himself, however, considerably relieved from the violence of the spasms after the first ten minutes. In twenty his strength failed; he was therefore raised from the bath, well rubbed, and laid in dry blankets.

The benefit derived from this experiment, was a temporary alleviation in the severity of the symptoms. It was proposed to try the effect of mercury, the ointment of which was several times rubbed in over the spine and limbs. The spasms were now universal, and probably from the irritation being constant, the spasmodic action was constant also, or rather the contractions returned in so rapid a succession, as to keep the whole frame in unceasing and terrible action; while the poor creature at times grinding his teeth, was constantly moaning from the excessive violence of the pain.

In the middle of the day after he had used the bath, the pulse was even, quick, and softer than before. On the next day (the 9th), the frequency of the spasms, and the horrible torments they produced, continued to increase upon him.

Towards the evening the spasms became more and more severe in their effects upon the organs of respiration, and at length he expired, apparently at the time when the same spasmodic action seized upon the heart.

In this case, the only point in the treatment which may be considered to have promised any thing, was that of introducing into the system a sufficient quantity of mercury to alter the prevailing tone and disposition of the nervous system. The frictions did not in this experiment produce any decided effect upon the mouth; but in other cases, in which the most satisfactory proofs of the full action of the remedy have existed, it has been found to have no power to arrest the progress of this most formidable disease.

### CASE II.

*In which Lock Jaw, attended with Tetanus, ended fatally within a Period uncommonly short.*

W. Richey, an Italian, aged 30, seaman on board his Majesty's ship Bellerophon, lost his right arm, which was shot away by a heavy ball. Part of the upper extremity of the humerus remaining, it was removed soon after by an operation similar to that which is performed for amputation at the shoulder-joint. Unfortunately the limb had been carried away so close to the body, that it was not possible to make any provision for the formation of a round stump by a fold of the integuments. The muscular parts projected considerably beyond the edge of the skin, and neither pressure nor adhesive applications rendered much assistance in bringing it into form.

This man constantly said he felt but very little pain in or about the wound. On the 8th day he complained of stiffness and pain about the angles of the jaw, and these pains increased and extended their influence so rapidly, that he was with much difficulty raised up in his bed on the following morning, in order to his being dressed and shifted. There seemed by this time to be an established state of general rigidity affecting all the muscular parts of the body and limbs, which prevented him from all voluntary tary tary motion; while his being in any manner disturbed in his person, was constantly productive of aggravated pain.

On the next day (the 10th) he was worse, all his sufferings being greatly increased; in the evening, he had, with much difficulty, been raised up to drink some water; he returned the vessel to the nurse, after he had swallowed some of the fluid, without any apparent increase of pain, or other aggravation of his symptoms; he complained of no distress or new uneasiness; he laid himself back in the bed, and silently breathed his last, not even uttering the least sound expressive of disturbance.

In this case the progress of the disease was so rapid, that almost before any vigorous measures were adopted for his relief, the man was lost. Mercurial frictions were commenced upon, but only on the day before the evening on which his dissolution took place.

There is not a doubt, but that in this case also, the sudden and unexpected close of the scene was the consequence of a sudden spasm seizing upon the heart, and probably the organs of respiration; the most general mode of termination in this disease.

I am, &c.

JOHN HOWSHIP,

Member of the Royal College of Surgeons, London, and Assistant Surgeon of the 52d Regiment of Foot.

*Scarborough, Feb. 6, 1809.*

